# Blood Immunity and Blood Relationship

**Published:** 1906-06

**Authors:** 


### Reviews of Books.

**Blood Immunity and Blood Relationship.** By GEORGE H. F. NUTTALL, M.A., M.D. Pp. xii., 444. Cambridge: The University Press. 1904.

This work is a most valuable contribution to our knowledge of hæmatology, and represents an enormous amount of painstaking labour. The material for examination has been obtained from various parts of the globe, and some 2,000 tests have been made with fresh and dried specimens of blood. The inter-relationship of the vertebrates has been investigated by means of the precipitin test, a research that has given remarkable confirmation of the most recent statement of the genealogical relationships of the mammalia and other vertebrates. There is an excellent review of the work hitherto done relating to antibodies in general, antibodies other than precipitins, and precipitins; but the bulk of the book consists of the records of precipitin tests by Dr. Nuttall, Dr. Graham Smith, and Mr. Strangeways. For the general reader the section dealing with the practical application of the precipitin reaction in legal medicine, &c., is of great interest, and clearly indicates that the precipitin tests are to play a prominent part in legal medicine. Indications are not wanting that, in the near future, precipitin tests will be made use of in clinical medicine, in the examination of urine, &c. The arrangement of the book, its thoroughness, and the scientific method of the work command our admiration. The clear description of the technique and the bibliography alone makes this book essential to all workers in newer branches of blood research.

